# A new approach for the implementation of ergonomics in sonography to prevent work-related musculoskeletal disorders (ErgoSon)

**DOI:** 10.1186/s12995-025-00457-6

**Published:** 2025-04-07

**Authors:** Johannes Matthias Weimer, Bastian Dumancic, Julia Weinmann-Menke, Josefine Rombusch, Benjamin Ernst, Alexa Krambeck, Rejane Golbach, Eugen Topal, Christian Maurer-Grubinger, Carlotta Ille, David A. Groneberg, Christina Erbe, Daniela Ohlendorf, Fabian Holzgreve

**Affiliations:** 1 Rudolf Frey Learning Clinic, University Medical Center of the Johannes Gutenberg University Mainz, Mainz, Germany; 2 Department of Medicine I, University Medical Center of the Johannes Gutenberg University Mainz, Mainz, Germany; 3 Department of Orthodontics, Medical Center of the Johannes Gutenberg-University Mainz, Mainz, Germany; 4 Department of Otorhinolaryngology, University Medical Center Frankfurt, Frankfurt am Main, Germany; 5 Department of Biostatistics and Mathematical Modeling, University Hospital Frankfurt, Frankfurt am Main, Germany; 6 Institute of Occupational Medicine, Social Medicine and Environmental Medicine, Goethe University Frankfurt, Frankfurt am Main, Germany

**Keywords:** Musculoskeletal disorders, Nordic questionnaire, Kinematic analysis, RULA, Inertial sensors, Ultrasonography, DOPS

## Abstract

**Background:**

A substantial body of research has documented a high prevalence of neck, shoulder, wrist, and back pain among sonography users. However, the specific postures that contribute to these complaints have scarcely been systematically investigated, to date. This proposed study offers a novel method to record users’ body posture during sonography examinations kinematically and to survey the complaints of sonography users in various specialities. Using this data, well-founded ergonomic recommendations for the prevention of work-related musculoskeletal disorders (WRMSDs) will be developed.

**Methods:**

A minimum of 38 study participants across two groups (19 beginners; 19 experienced) per speciality (head and neck sonography, abdominal sonography, cardiac sonography, musculoskeletal sonography, and obstetric/gynaecological sonography) will be assessed using kinematic whole-body (including finger movements) analysis based on inertial motion capture. Subsequently, ergonomic risk will be determined by integrating the quantitative data into the Rapid Upper Limb Assessment (RULA). Moreover, a questionnaire on musculoskeletal complaints and ergonomics in sonography will be used in certified sonography courses, ultrasound-based centres, and university teaching. The primary outcome measures of this proposed study include typical tasks based on joint angles and assessment using RULA scores. In addition, the prevalence of WRMSDs will be recorded.

The Mann-Whitney-U test will be employed to calculate the differences between the two study groups in each speciality. In addition, inferential statistical comparisons will be conducted for continuous data using confidence bands; the statistical parametric mapping method will be employed here. The significance level will be set at *p* = 0.05.

**Conclusions:**

This article proposes a study (or series of studies) to describe the continuous ergonomic risk for typical tasks across different disciplines of sonography and to identify increased ergonomic risks. Such studies offer significant potential for preventing WRMSDs. The insights gained could inform the future design of prevention programmes and the development of recommendations for action, as well as teaching sonography users an ergonomically optimised way of working. The results could suggest that ergonomics training is incorporated more thoroughly into ultrasound training curricula to minimise health risks for future users.

**Supplementary Information:**

The online version contains supplementary material available at 10.1186/s12995-025-00457-6.

## Background

Ultrasound diagnostics, a radiation-free, rapidly available, non-invasive examination method, have become important in many medical specialties in recent years, particularly with the advancement of technical capabilities [1]. Sonography is clinically conducted by medical professionals except in certain countries where specially trained sonographers are employed [2]. During the examination, the examiner typically assumes a seated position on a chair while the patient is positioned either recumbent or in a seated posture on a bed. The transducer is guided by the operator with either the right or left arm and pressure is applied as needed. The operator’s contralateral hand operates the device. The time required for an ultrasound examination varies significantly between one and thirty minutes depending on the type of examination and the specific clinical question being addressed [3]. For example, focused assessments such as evaluating the thyroid or salivary glands typically take 5–10 min. In contrast, abdominal sonography, which involves examining multiple organs like the liver, kidneys, and pancreas, can take 20–40 min depending on the complexity and the patient’s condition. Similarly, cardiac ultrasound (echocardiography), used to assess heart function and structure, often requires 20–30 min for a detailed evaluation. Musculoskeletal ultrasound, which examines joints, tendons, or soft tissues, varies widely, with simpler assessments taking 10–15 min, while more complex joint evaluations may require up to 30 min [4].

Perhaps unsurprisingly in this context, musculoskeletal disorders among sonographers are prevalent. Zhang et al. [5] reports a 12-month prevalence affecting 95% of their respondents with neck pain, 84% with right shoulder pain, 81% with right wrist/hand pain, and 82.4% with lower back pain. This frequency is perhaps explained by the existence of several proven risk factors for the prevalence of musculoskeletal diseases (MSDs) in the typical practice of sonography examinations, including the continuous performance and high required frequency of examinations, the associated assumption of identical body postures (a stress type of the German Social Accident Insurance’s (DGUV) ‘activities with forced body postures’), and the limited breaks between examinations [6–15]. In addition, patient comfort and the acquisition of optimal ultrasound images frequently supersede considerations of work posture and this can contribute to an increased prevalence of work-related musculoskeletal diseases WRMSDs [16].

Work-related musculoskeletal diseases and the occupational limitations caused by such diseases are typically investigated in the current literature through surveys of user groups, with variants of the Nordic Questionnaire recording the current status of musculoskeletal diseases [17–19]. The incidence of WRMSDs has been documented extensively in various clinical contexts [5–7, 11, 13, 14, 17, 18, 20–24]. Similar results to Zhang et al.’s have been observed in other studies that have surveyed cardiac sonographers, hospital ultrasound sonographers, practitioners of obstetric and gynaecological ultrasound, and veterinary echocardiographers [6, 11, 14, 17, 23, 25].

For the prevention of WRMSDs, ergonomic factors remain a significant area of interest and investigation across numerous professional disciplines, including in medical sonography [24, 26–28]. Prior studies and professional guidelines recommend that, in addition to training in technical knowledge, users should also be taught aspects of ergonomics to prevent WRMSDs [11, 29–31]. Still, relatively little scholarly attention is devoted to the domain of ergonomics in the workplace [32, 33], with recent studies highlighting the continuing need for further research in this area [11, 18, 22, 34–36]. Furthermore, there is a need for extended and more detailed recommendations regarding ergonomic improvements from the perspective of the sonography users of different user groups [33, 37].

As with other WRMSD research, current research on the ergonomics of ultrasound diagnostics has been primarily based on surveys and interviews with sonography users and has led to the identification of key areas for improvement [6, 7, 12, 13, 17, 18, 20, 24–28, 34]. Nevertheless, the existing literature offers only limited guidance on specific measures to prevent WRMSDs or to improve ergonomic conditions in the sonography workspace [10, 11, 37, 38]. International expert groups and professional societies such as the American Institute of Ultrasound in Medicine (AIUM), in collaboration with other organisations, recommend that a structured optimisation of the ultrasound examination setting should be undertaken, to include the use of height-adjustable tables, chairs and screens on sonography devices, as well as changes to the movement sequences with regard to transducer guidance and a more ergonomic sitting position [10, 11, 37, 38].

Few studies go beyond questionnaires and ergonomic recommendations, with very few using quantitative kinematic data to generate insights [39–42]. Nevertheless, this is a promising area of research. Emerging technologies in motion analysis now enable the precise capture and reconstruction of movement patterns under real-world working conditions. These data can be integrated into an ergonomic assessment system, providing continuous scoring for all relevant joints and movements [39–42]. This approach facilitates the objective quantification and targeted analysis of ergonomic stress, a methodology that has already been successfully applied in other professional fields [40, 43–49]. In addition, this approach could help to objectively quantify previously described risk factors, identify further risk factors, and refine or extend previous recommendations.

## Aims

For the first time, it is now possible to precisely record actual movement sequences during ultrasound examinations. Our proposal suggests collecting and analysing data pertaining to WRMSDs and ergonomics during a standardised ultrasound examination [50, 51]. Kinematic data will be gathered from sonography users using an MVN Awinda motion capture measuring instrument from Movella (Movella Holdings Inc., Enschede, Netherlands). The data obtained will be integrated into an ergonomic evaluation system that allows continuous scoring of all relevant joints and movements. Established ergonomic risk assessment tools (ERATs) were used to systematically analyse and evaluate the ergonomic conditions [52–57]. The integration of kinematic data into established ERATs represents a novel approach that offers the potential for an objective and continuous assessment of ergonomic risk [42, 58]. The combination of data collected by inertial systems and the Rapid Upper Limb Assessment (RULA) method has already yielded promising results in other contexts [42, 44, 45, 59]. Mauer-Grubinger et al. [42] have developed a script for this purpose that has already been successfully employed in the context of dentists and office workplaces [44, 45, 60, 61]. We propose to deploy it for the first time in an ultrasound context.

The Maurer-Grubinger method combines the observational strengths of RULA with the precision of kinematic analysis provided by inertial motion capture (IMU) systems. This hybrid model calculates ergonomic scores for multiple body regions over time, offering a flexible, automated tool for assessing and mitigating workplace ergonomic risks. This approach enhances RULA by IMU data, allowing for dynamic, high-resolution analysis of ergonomic risks. Unlike traditional RULA, which relies on static posture observations, this method provides continuous data on joint angles and movements throughout a task cycle. This improves sensitivity, reduces observer bias, and captures nuanced postural variations that might otherwise go unnoticed. By combining observational and kinematic methods, the approach refines risk assessment and offers a more detailed foundation for workplace interventions, making it a significant advancement over standard methodologies.

We apply the methodology developed by Maurer-Grubinger et al. [42] with a view to identifying relevant occupational movement patterns in the field of sonography.

The following objectives were formulated as part of this proposed project:

- A questionnaire will gather information on the arrangement of examination inventory in ultrasound centres and clinics (such as the examination bed, sonography device, and the examiner’s chair) to conduct an initial ergonomic and ratio-based analysis.
- This proposed study will include doctors, students, and other medical staff (such as paramedics and physician assistants) from a wide range of disciplines and training levels who are enrolled in certified sonography courses, including those accredited by professional societies across basic, advanced, and master-level courses [62], as well as participants from ultrasound centers and university teaching programs. All participants will be required to complete a questionnaire to assess the following variables: age, height, weight, experience with sonography (number of examinations performed at previous, regular screenings per month), and knowledge about ergonomic working practices. Furthermore, relevant WRMSD factors for the clinicians and in the clinical context will be identified (such as height-adjustable features in the treatment setting and their utilisation, musculoskeletal system complaints using a modified Nordic questionnaire, and the potential contribution of work and private activities to MSDs). Finally, current complaints when performing a sonography will be documented (before, during continuous ergonomic assessment, and after the scan).
- Direct Observation of Procedural Skills (DOPS) tests will identify and describe typical movement profiles in sonography and subsequent continuous ergonomic assessment (RULA) of all relevant joints and movements. These tests will be organised according to the body region/organ systems, namely: (1) neck (glands and soft tissue assessment), (2) musculoskeletal regions (shoulder, knee, ankle), (3) abdomen (upper abdomen and lower abdomen), (4) thorax (heart and lungs), (5) gynaecology/obstetrics (transvaginal assessment and transabdominal assessment of pelvic organs/pregnancy [on a simulator]), and (6) vessels (leg veins, abdominal vessels and neck vessels).
- The ergonomic risk will then be assessed based on the practitioner’s professional experience, categorised into two experience levels—beginner and experienced—within their respective speciality.

## Methods

### Approach process

The approach will be divided into three steps. Step 1 will use a questionnaire to analyse the ergonomic setup in ultrasound centres (e.g., equipment arrangement) and to collect data on participants’ demographics, ultrasound experience, ergonomic practices and musculoskeletal complaints. Step 2 will assess typical sonographic tasks using inertial motion capture in a DOPS test across different body regions and disciplines. Step 3 will assess ergonomic risks using RULA, focusing on joints and movements, and categorised by practitioner experience level (beginner vs. experienced).

### Subjects

Measurements will be conducted with participants from the head and neck sonography, abdominal sonography, cardiac sonography, musculoskeletal sonography, and gynaecological sonography specialisms. The questionnaire will establish competence groups (beginners and experienced) for each speciality, with the sonography sequence recorded for a minimum of 38 participants per speciality, according to experience (19 each). The categorisation as ‘experienced’ is based on current certification guidelines of professional associations.

The study population will comprise women and men between the ages of 18 and 70 who perform sonographies in the aforementioned areas and operate the ultrasound probe with the right hand, except for echography, where the ultrasound probe is also guided with the left hand. Individuals with recent injuries (herniated discs, spinal injuries), rheumatic diseases, severely restrictive deformities (scoliosis) of the spine or stiffened spinal joints (pathological or surgically induced), or genetic muscle diseases will be excluded from participation.

The study approach has been approved by the Ethics Committee of the Department of Medicine of Goethe-University Frankfurt (115/23).

### Recruitment

The study approach will include doctors, students, and other medical staff (paramedics, physician assistants) from a wide range of disciplines and levels of training from certified sonography courses and ultrasound centres, as well as university teaching.

Questionnaires will be distributed at ultrasound centres. A representative cross-sectional sample of the doctors who respond to the questionnaire will be selected as subjects for the study.

### Measurement protocol

#### Step 1: online survey

The online questionnaire will comprise items derived from the questionnaire on musculoskeletal complaints (FB*MSB) developed by the Federal Institute for Occupational Safety and Health (BAuA) [63]. This will be consistent with the updated German iteration of the Nordic Questionnaire, as proposed by Kuorinka et al. [64]. The questionnaire (FB*MSB) will include items on the prevalence of musculoskeletal complaints in specific body regions over the past 7 days, in the past 4 weeks, and in the past 12 months. These regions include the neck and cervical spine, shoulder joints and upper arms, elbows and forearms, hands and wrists, thoracic spine, lumbar spine and lower back, hip joints and thighs, knee joints, lower legs, and feet and ankles. The names of the ten body regions correspond to those used in the BAuA questionnaire on musculoskeletal complaints (FB*MSB) [63]. The BAuA version employs corresponding illustrations.

In addition to a survey of baseline characteristics, including position, age, gender, height, weight, handedness, degree programme/semester of study or specialisation/year of further training at German Society for Ultrasound in Medicine (DEGUM), the questionnaire will contain items specific to sonography. These include previous experience in sonography (number of independent examinations, participation in ultrasound courses, time spent in ultrasound diagnostics), current activity in the field of ultrasound (frequency of use of sonography in everyday clinical practice, transducer handling, examination position), and previous experience in ultrasound ergonomics (general aspects of ergonomics, attendance of training courses on ergonomics at the workplace). The questionnaire will also address ergonomic aspects of ultrasound examinations, including the current status of ergonomics in the workplace. This section enquires about the values placed on ergonomically correct working, ergonomic measures taken by employers, the ergonomic setup of the sonography workplace, and the adaptation of the workplace during ultrasound examinations.

Finally, the questionnaire will address motivation and the need for ultrasound ergonomics. It will ask respondents to consider the relevance of the topic, their motivation for training, ergonomic challenges and suggestions for improvement.

The questionnaire will be constructed online via the “LimeSurvey” platform and in accordance with the recommendations of Checklist for Reporting Results of Internet E-Surveys (CHERRIES) [65].

#### Step 2: motion capturing and DOPS

**Fig. 1:**
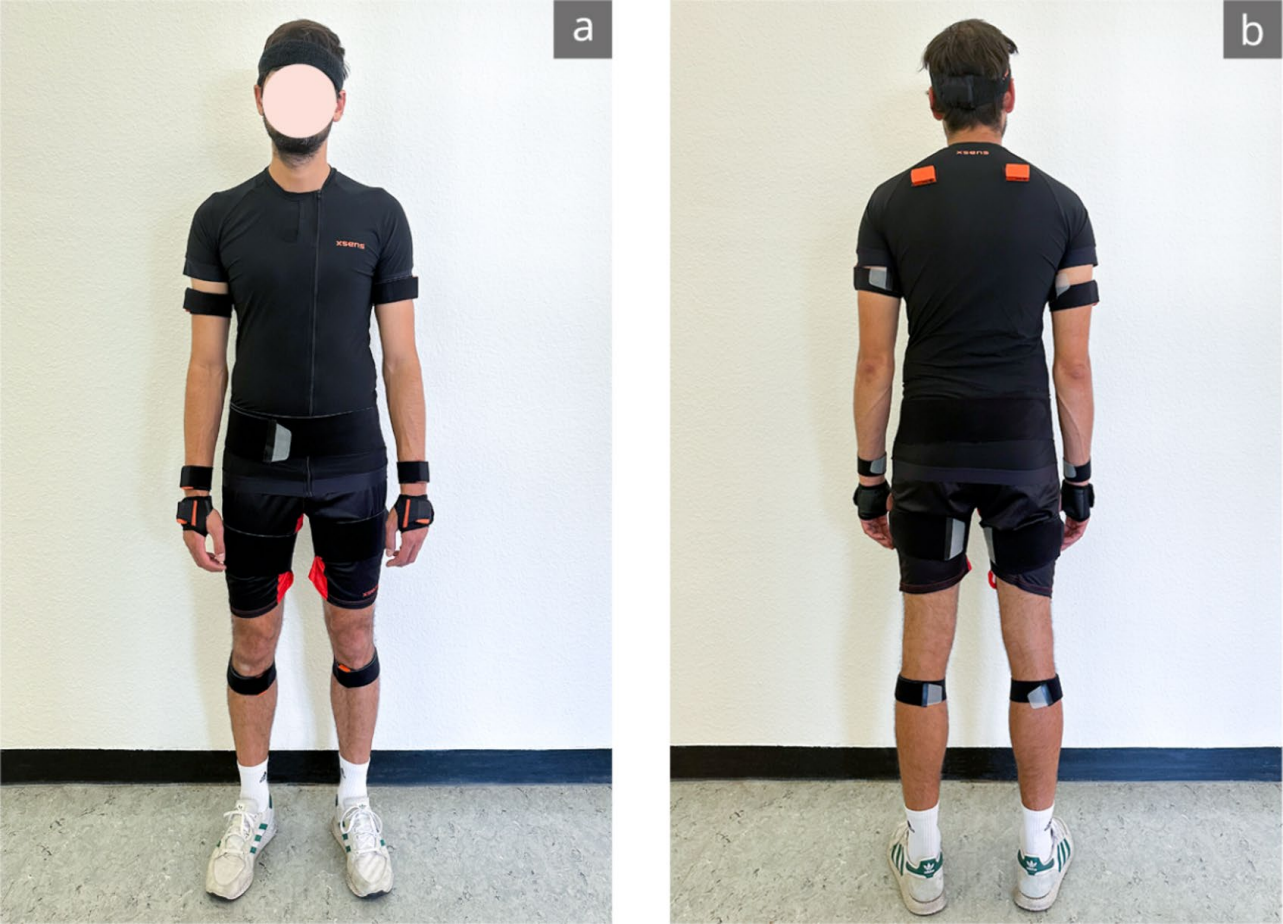
The Awinda setup comprises the attachment of 17 sensors which are secured with straps to the relevant body segments. View of the subject from ventral (**a**) and dorsal (**b**). The foot sensors were attached under the shoe tongue

The recording of posture will be carried out by means of the inertial motion capture system MVN Awinda from Movella (Movella Holdings Inc., Enschede, Netherlands). The system measures 22 joint angles in all three degrees of freedom at a frequency of 60 Hz, resulting in highly precise motion data throughout the entire workflow. Fundamentally, the system comprises 17 IMUs containing accelerometers, gyroscopes, magnetometers and barometric altimeters (Fig. 1). MVN Awinda utilises sophisticated algorithms and signal processing techniques to interpret the motion data captured by the IMUs. Movements of the sensor wearer are accurately reconstructed in real-time, providing immediate feedback and visualisation of the motion sequences. The alignment error for angular velocity, acceleration and the magnetic field is specified by the manufacturer as 0.1 deg [66].

The analysis of hand and finger movements is derived from the Xsens Metagloves, developed by Manus Meta (Manus Meta, Geldrop, Netherlands), which enable precise and intuitive tracking based on magnet field tracking, with a sampling rate of 120 Hz and a signal latency of ≤ 7.5 ms. The sensors are strategically placed across the hand and fingers within the glove. Each sensor within the glove includes accelerometers, gyroscopes and magnetometers to capture the acceleration, rotation and orientation of the hand and fingers in all three degrees of freedom, enabling the precise tracking and reproduction of the hand movements and gestures. The Xsens Metagloves are integrated into the Awinda system and its associated software platforms.

After the sensors haves been attached and calibrated the recordings can then take place over a period of eight minutes for each DOPS. It is only the “hard skills” of the objective structured assessment of ultrasound skills (OSAUS) scale applied or modified in the DOPS that will be considered in relation to the “examination performance/systematic examination” [50, 51, 67]. These include the adjustment/assessment of various organs/body regions according to structured orientation sections. Table 1 lists the different organ systems/regions that should be examined in the DOPS.

**Table 1:** List of the various applications of ultrasound imaging, including the specific organ systems and regions to which they pertain

Topic	Organ systems/region
Head and neck sonography	• Gland/soft tissue assessment
Musculoskeletal sonography	• Shoulder • Knee • Ankle joint
Abdominal sonography	• Upper abdomen • Lower abdomen
Thoracic sonography	• Heart • Lung
Gynaecological and obstetric sonography	• Transvaginal assessment • Transabdominal assessment
Vascular sonography	• Leg veins • Abdominal vessels • Neck vessels

**Fig. 2:**
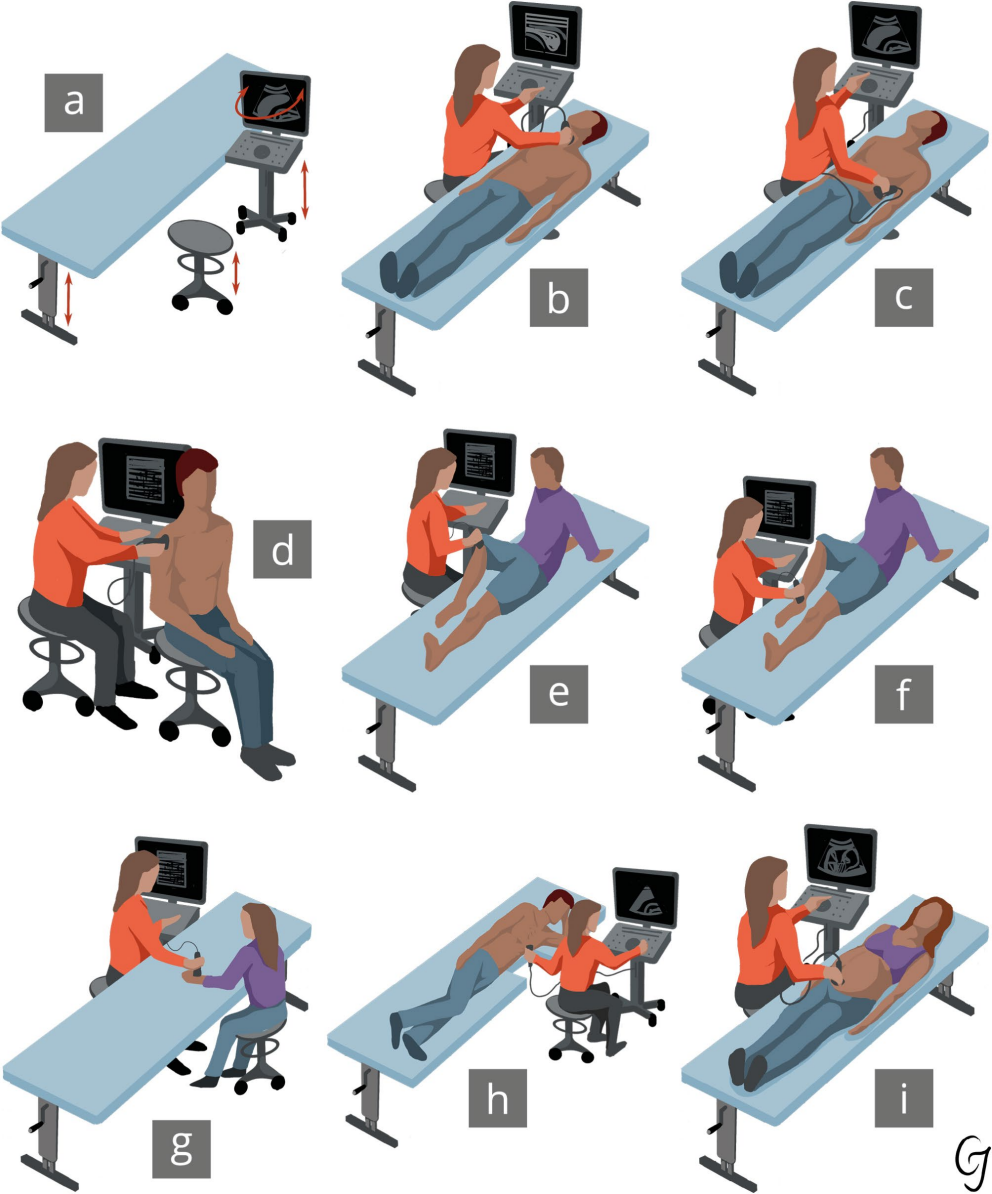
Presentation of the fields of application of sonography. The material equipment (**a**) as well as the performance of a sonography of the head and neck (**b**), the abdomen (**c**), the shoulder (**d**), the hand (**e**), the knee (**f**), the foot (**g**), the heart (**h**) and a pregnant woman (**i**) is shown

The ergonomic risk analysis of body postures when performing a sonography in the form of a DOPS is carried out according to the body region/organ systems (Fig. 2). The regions are as follows: (1) neck (glands and soft tissue assessment), (2) musculoskeletal regions (shoulder, knee, ankle), and (3) abdominal region, subdivided into upper and lower segments; the thoracic region encompasses the heart and lungs, while the gynaecological and obstetric examination includes transvaginal and transabdominal assessments of the pelvic organs and pregnancy that are conducted on a simulator. Finally, (4) the vascular system examination encompasses the examination of the lower extremity veins, abdominal vessels and cervical vessels.

To thus ensure the precise allocation of activities, the entire measurement sequence will be filmed in a single, comprehensive view (iPad Air, Apple Inc., Cupertino, California, United States) with a resolution of 1080p HD at a frame rate of 120 fps. The measurement system and the camera will be synchronised by means of the software. Due to the character of the work, no randomisation of the tasks will be carried out.

#### Step 3: rapid upper limb assessment (RULA) and data processing

**Fig. 3:**
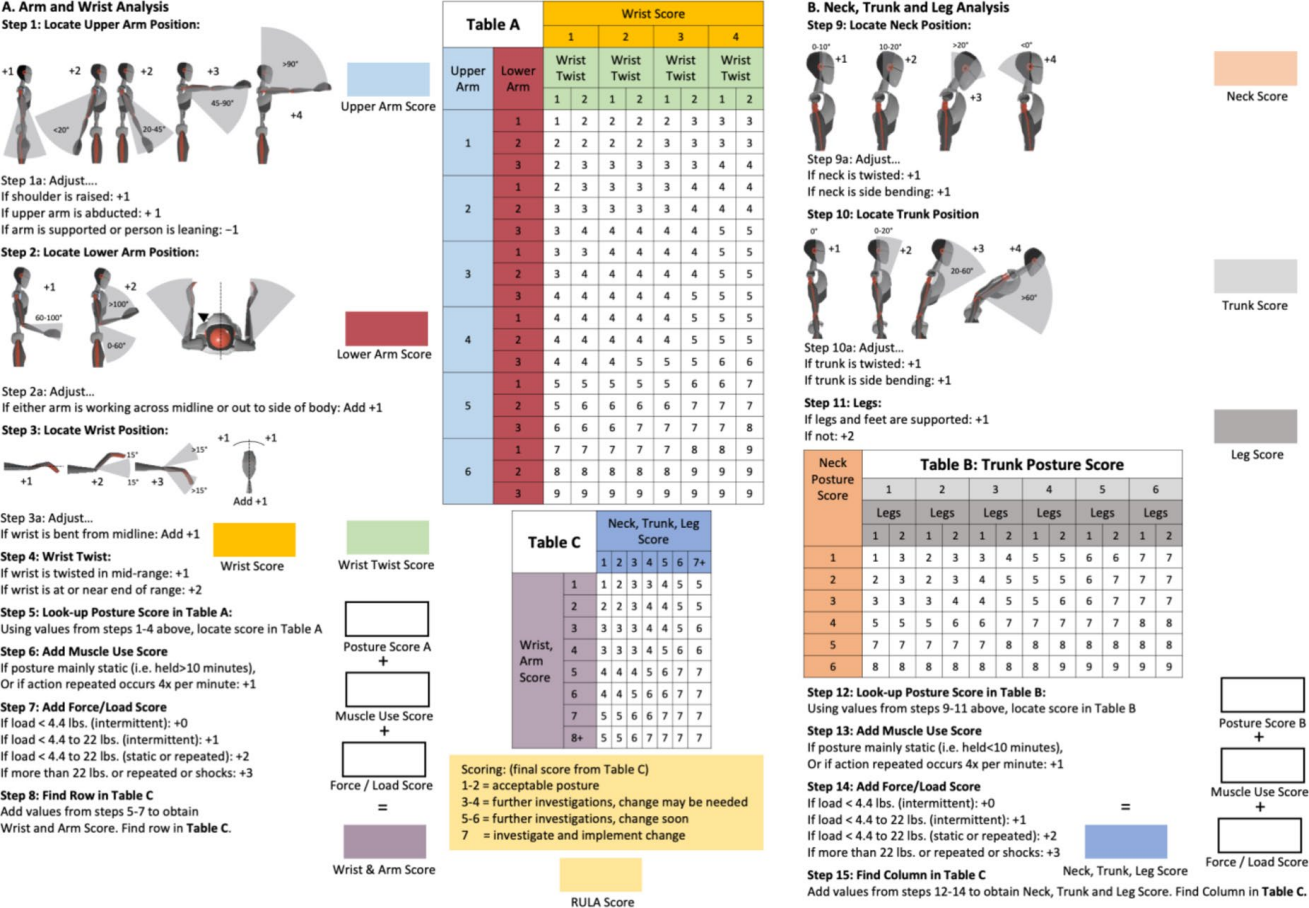
RULA worksheet showing all relevant RULA steps accumulating the final RULA score

McAtamney et al. [68] initially devised the Rapid Upper Limb Assessment (RULA) in 1993 with the objective of evaluating musculoskeletal strain in occupational settings where work-related upper limb disorders are prevalent. No sophisticated equipment is necessary for this assessment. The assessment can be completed rapidly and provides valuable insights into an individual’s posture, muscle function and the external load they experience. Furthermore, a scoring system is provided to offer an overview of the strain on individual body parts (Fig. 3).

To integrate RULA into the objective, continuous data of the IMU system, all RULA limits must be defined quantitatively. The RULA protocol has, thus, been adapted for this purpose. The integration of inertial data into RULA has already been published and is to be applied in this approach [42]. A single RULA evaluation is obtained per pixel (60 Hz), thereby ensuring the continuous analysis of ergonomic load over the entire measurement period [42].

As the angles calculated by the IMUs are the actual measured joint angles, it is possible to exclude parallax errors that can occur in classic 2D images or videos.

### Evaluation criteria

1. The endpoints of the questionnaire will mainly be collected through selection options (nominal scales) or ordinal scaled items. In some cases, open answers will also be possible.
2. From the data of the biomechanical analysis, the risk score will be determined using the Rapid Upper Limb Assessment (RULA) [42].

The target values below have been derived from the resulting RULA scores to differentially assess the ergonomic risk. This has been done for both the overall RULA score and for the individual, body-regional RULA scores of the test subjects:

Kinematic/ergonomic outcomes:

- The median and interquartile range (IQR) of the total RULA analyses per pixel are presented.
- The relative average risk value over time (Rel. av. RST) is defined as follows:

The median and interquartile range (IQR) of the total RULA evaluations per pixel will be presented.

The following formula was employed to define the Rel. av. RST:

$$\begin{aligned} \:&Relative\:time\:spent\:in\:RULA\:score\:1\:\times\:\:1\:\\&+\:relative\:time\:spent\:in\:RULA\:score\:2\:\times\:\:2\:\\&+\:relative\:time\:spent\:in\:RULA\:score\:3\:\times\:\:3\:(\cdots)\:\\&+\:relative\:time\:spent\:in\:RULA\:score\:7\:\times\:\:7 \end{aligned}$$

RULA total score (“Final overall”): this includes the body parts from the right or left with the greater risk in each case.

RULA—total score on the right (“Final overall right”): this looks at the right half of the body (Step 1 right, Step 2 right, Step 3 + 4 right, Step 9, Step 10).

RULA—total score on the left (“Final overall left”): this considers the left half of the body (Step 1 left, Step 2 left, Step 3 + 4 left, Step 9, Step 10).

c. Local scores for a) and b):
  Upper Arm Score (left and right) - RULA Step 1
  Lower Arm Score (left and right) - RULA Step 2
  Wrist Score (left and right) - RULA Steps 3 + 4
  Neck Score - RULA Step 9
  Trunk Score - RULA Step 10
d. The joint angles of all three lines of freedom of the 22 recorded joints will be measured. The joint angles will be plotted against the frequency of occurrence.

To date, there are no automated procedures for the ergonomic evaluation of inertial data on finger movement. Accordingly, the joint angles of the various finger joints and their position and dispersion measures are the primary focus of analysis. Furthermore, static or repetitive periods can be defined for the recorded finger joints based on the angular acceleration.

### Statistical data analysis

#### Power analysis

The power analysis refers to the statistical comparison between the groups (beginners vs. experienced), as p-values are recorded here. A total of 38 participants (19 per group) are to be included in the proposed study. The sample size calculation was carried out in collaboration with the Institute for Biostatistics and Mathematical Modeling at the University Hospital Frankfurt, Goethe University, and is based on the results of the comparison of orthodontists with endodontologists from study Holzgreve et al. [44]. A Mann-Whitney estimator (which describes the probability that a value from group 1 is smaller than a value from group 2) of 0.2311 was calculated. For the comparison of the two groups (beginner and experienced), an effect at least as large is expected. Since a normal distribution of the measurements from the two samples cannot be assumed, the sample size calculation is based on a Wilcoxon-Mann-Whitney U test. The sample size of the entire study population (n1 + n2) is realistically set at *n* = 38, with a power of 80% and α = 0.05. The sample size calculation was performed using BiAS for Windows, and dropouts are not expected due to the study design.

#### Evaluation of questionnaire

The analysis will be conducted exclusively on the basis of fully completed questionnaires. The data will be prepared using Microsoft Excel. The questionnaires will be subjected to a plausibility check to ascertain the consistency of the responses provided. The questionnaires will be analysed using IBM SPSS Statistics 29 (IBM, Armonk, NY, USA), and the results presented in a descriptive manner, broken down according to the participant’s professional experience levels (beginners and experienced). Corresponding measures of position and dispersion will be calculated to achieve this. The socio-demographic data will be calculated separately for both level of professional experience. To ascertain whether the subject data, which will be presented with location and dispersion measures, is normally distributed, the Kolmogorov-Smirnov-Lilliefors test will be carried out.

#### Baseline group comparisons

The Mann-Whitney-U test will be employed to ascertain the disparities between the categories of work experience for the outcome variable RULA (evaluation criteria 2).

Furthermore, inferential statistical comparisons are conducted for continuous data of the joint data (evaluation criteria 3) with confidence bands. The statistical parametric mapping (SPM) method [69] will be employed to achieve this.

The level of statistical significance will be set at α = 5%.

## Discussion

The objective of this proposed study is to analyse the ergonomic conditions and WRMSD risks inherent to sonography through a combination of preliminary interviews with sonography users and the use of a motion capture system to quantify data. The study will endeavour to provide and evaluate training recommendations as well as ergonomic recommendations.

In the absence of detailed information regarding the configuration of a given clinical context’s equipment inventory, it will be essential to undertake an initial ergonomic and ratio-related analysis of ultrasound examination equipment in centres based on the photographic evidence. In particular, the criteria of the arrangement and height adjustability of the examination bed or chair, the sonography device, and the examiner’s chair form the focus of attention. A questionnaire will be completed by all sonography users, providing information on age, height, weight, sonography experience and ergonomic knowledge. The occurrence of musculoskeletal complaints will be recorded using a modified Nordic Questionnaire. An ergonomic risk analysis of data gathered during DOPS tests will encompass the postures adopted during sonography of the neck, musculoskeletal system, abdomen, thorax, gynaecology and vessels.

Following the data collection phase, a comparison of the ergonomic risks will be conducted according to the professional experience levels of the participants, categorised as ‘beginner’ or ‘experienced’ across the various specialities.

A comprehensive analysis will be conducted on the demographic and ergonomic data of the sonography users, along with an in-depth examination of the specific body postures adopted during various ultrasound procedures. The findings will help inform the development of preventive measures and the formulation of generally applicable recommendations for the ergonomic performance of sonography. To the best of our knowledge, no previous research has adopted this approach and subsequently published the findings. Should the ergonomic risk calculations indicate the necessity for action in accordance with the prevailing literature, behavioural and situational preventive measures will be developed or further developed. Such measures include, for instance, the implementation of ergonomic training programmes with the objective of reducing ergonomic risk.

The results of recent surveys indicate that work at sonography workstations is predominantly characterised by unfavourable static postures. These postures can lead to musculoskeletal complaints from users in their neck, shoulders, hand (joint), or back area [5–7, 11, 13, 14, 17, 18, 20–24]. Such poor posture is also a general risk factor for work-related musculoskeletal disorders in other workplaces [70].

It is therefore evident that the current issues identified by sonographers and other users of sonography [11, 18, 22, 34–36] should be the primary focus of current research endeavours. The methodology described in this proposed study can, thus, provide valuable insights by not only confirming the problems subjectively reported in the questionnaires, but also objectively substantiating them based on quantitative data. Furthermore, the frequency of use (daily, weekly, monthly) can be ascertained, thus enabling the derivation of individual risk profiles.

By analysing this quantitative and qualitative data on WRMSDs and ergonomics, it may be possible to derive recommendations for the performance of ergonomically correct sonography that could be considered broadly applicable. Existing recommendations, as exemplified by those from the AIUM [71] could be expanded and modified to be tailored even more closely to the specific applications in question (e.g. to those of non-sonographers or doctors).

The RULA method may be employed for the assessment of ergonomic risks and would permit the comprehensive analysis of the work processes. In contrast with manually completed observation methods that provide only a subjective account, the integration of kinematic movement data in RULA enables an objective risk assessment for each recorded pixel. This method was previously published by Maurer-Grubinger et al. [42] in 2021, compared to the conventional RULA application used by Nowara et al. [72] in 2023, and was successfully applied and further developed in the SOPEZ project [43, 44, 46–48]. This refined methodology will provide a foundation for advancing our understanding of the risks associated with MSDs in sonography.

A further advantage of the RULA method is that it considers both the static positions of the trunk and proximal upper extremities, as well as the repetitive movements of the hands that also represent a risk for MSDs [5]. A review of the literature reveals that surveys conducted in the past among sonography users have also demonstrated a high prevalence of musculoskeletal disorders affecting the wrists and hands [5].

Despite the existing literature on the ergonomic risks faced by ultrasound users, there is still a dearth of dedicated measurements that objectively represent the arrangement of the ultrasound device and the positioning/posture of the user. Furthermore, a comparison of the fields of specialisation and the levels of experience is likely to provide insight into the phase of work experience in which unfavourable postures may occur. As demonstrated by the questionnaire analyses conducted by Finsen et al. [73], the prevalence of MSDs is observed to increase with the accumulation of work experience.

As the described measurement methodology of ergonomic analysis based on quantitative data collection using IMUs has not yet been employed in any sonography application, the data and analyses obtained could contribute to the expansion of the field of ultrasound ergonomics and the establishment of new, supplementary standards. It is recommended that these new standards and the general topic of ‘sonography ergonomics’ be more strongly integrated into current training curricula in the future.

### Limitations

Recording the entire ergonomic exposure during the sonographic examination inevitably also requires measuring the force exerted by the hands. However, it is not yet possible to reliably perform these measurements during sonography. We are currently working on the validation of smart gloves to overcome this limitation. In the medium term, the aim is to establish the recording of pressure loads using pressure sensors integrated into the textile of the smart gloves to enable a more precise analysis of manual loads.

A potential self-selection bias represents a limitation of the planned studies, both in the ergonomic analyses and in the questionnaire survey. Participants will probably be recruited in sonography courses, medical practices, hospitals, and via specialist organisations and clinics that carry out ultrasound examinations. In all cases, the decision to participate is at the discretion of the people contacted, which could mean that people with a particular interest in ergonomic issues or pre-existing musculoskeletal complaints are more likely to participate.

Random selection of respondents is difficult to implement in this setting, which limits the possibility of achieving a representative sample. This could affect the generalisability of the results of both the ergonomic analyses and the survey results. This limitation should be carefully considered when interpreting the results of the planned studies. Furthermore, as the measurements will be conducted in laboratory settings, there is the possibility that the external validity of the findings may be limited. It should be noted that the recordings will not be carried out in a person’s own practice and, therefore, this may result in the omission of certain routine work processes. The use of DOPS serves to enhance the standardisation of the work processes. The RULA method may lack sufficient sensitivity when differentiating between various conditions due to the coarse categorisation of the thresholds and the presumed low variability of movements in the sonography domain. Conversely, an analysis based on joint angles may identify the most subtle differences, although this would not facilitate an ergonomic classification.

While recognizing the value of EMG cuff measurements, the study team has chosen to exclude them due to a focus on kinematic analysis aligning with the study’s primary aim of investigating sonography user postures. The use of EMG would complicate the methodology, potentially affect participant behavior, and require additional resources, making it less relevant to the postural ergonomics focus of the research. The combination of kinematic data with the Rapid Upper Limb Assessment (RULA) is deemed sufficient for ergonomic risk assessment within the study’s scope.

### Future research

The high prevalence of WRMSDs in sonographers, as evidenced by numerous studies, underscores the need for modifications in sonography practice. For these recommendations to be implemented in a targeted manner, it is first necessary to collect quantitative data on posture during sonography examinations. The data obtained in this project from the ergonomic risk analysis of posture during sonography examinations will help to inform the derivation of behavioural and situational preventive measures. Subsequently, the data collected may be employed in future studies to analyse the ergonomic aspects of diverse sonography arrangements. The results of the planned project could also be useful for other medical fields and could be transferred if necessary. These include, for example, endosonography, endoscopic techniques, and special surgical procedures. Future research could further investigate ergonomic training as an avenue for practical application of the studies’ results. If the conducted ergonomic analyses indicate a need for ergonomic improvements, a customised ergonomic training programme could be developed. This should aim to promote low-risk postural profiles and optimise ergonomic practices during sonographic examinations. This could focus on specific areas such as posture and guidance of the ultrasound probe as well as positioning in relation to the patient and device.

One possible implementation would be the integration of such training into the training programme for sonography users, for example with a blended learning approach. Moreover, a substantial body of evidence from numerous studies indicates that training interventions, including strength training and stretching, can markedly reduce the prevalence of WRMSDs [74–77]. A comparison of different intervention categories has revealed that strength training is particularly effective in reducing upper extremity MSDs as part of workplace health interventions [74]. The AUIM recommendations also favour the implementation of preventive measures [11] and emphasise the necessity for strategies to measure and record WRMSDs. Furthermore, it would be beneficial to place a stronger emphasis on ongoing regular education in the future [78].

The proposed project aims to conduct a continuous ergonomic risk analysis for typical sonographic activities to better understand the ergonomic risks of specific work processes, as no such profile yet exists. Still, the question of the extent to which different combinations of tasks and breaks influence the ergonomic risk requires individual consideration. A detailed ergonomic assessment of the workload caused by different tasks is essential for this and will be carried out systematically as part of this project. The development of a tool that considers, for example, the individual workload of sonographers including working hours and break times should be the subject of future research. Future work should also focus on potential cost and access barriers to the adoption of advanced ergonomic technologies in sonography practices to realise practicable recommendations.

## Conclusion

The aim of this proposed study is to create kinematic motion profiles for typical tasks in different disciplines of sonography and to identify increased ergonomic risks. The described method offers a novel approach to collect and expand important aspects and data on ergonomics in sonography using motion analysis, an area that has been little studied so far. From a preventive perspective, there is considerable potential for avoiding work-related musculoskeletal disorders (WRMSDs) in sonographers. The knowledge gained can support the future design of prevention programmes, the development of recommendations for action, and the teaching of ergonomically optimised working methods for sonographers. In addition, there is an urgent need to integrate the topic of ergonomics more strongly into training curricula to minimise health risks for current and future users.

## Supplementary Information

Supplementary Material 1.

## Data Availability

No datasets were generated or analysed during the current study.

## Abbreviations

DGUV: German Social Accident Insurance
MSDs: Musculoskeletal diseases
WRMSDs: Work-related musculoskeletal diseases
AIUM: American Institute of Ultrasound in Medicine
ERATs: Ergonomic risk assessment tools
RULA: Rapid upper limb assessment
IMU: Inertial measurement units
DOPS: Direct observation of procedural skills
FB*MSB: Questionnaire on musculoskeletal complaints
BAuA: Federal Institute for Occupational Safety and Health
DEGUM: German Society for Ultrasound in Medicine
Cherries: Checklist for Reporting Results of Internet E-Surveys
OSAUS: Objective structured assessment of ultrasound skills
IQR: Interquartile range
Rel. av. RST: Relative average risk score
