# Supplementary material for: A new approach for the implementation of ergonomics in sonography to prevent work-related musculoskeletal disorders (ErgoSon)
Source: J Occup Med Toxicol. 2025 Apr 7;20:11. doi: 10.1186/s12995-025-00457-6 (PMC11978026; doi:10.1186/s12995-025-00457-6)
Supplement: Supplementary file 1 — Supplementary Material 1. [file 12995_2025_457_MOESM1_ESM.pdf]

Section A: Introduction

A1. **Informed consent for the planned study:**

I have been thoroughly and clearly informed about the study and its objectives. All my questions have been answered to my satisfaction, and I was given the opportunity to ask additional questions. I am aware that I can withdraw my consent to participate in this study (either verbally or in writing) at any time and without having to provide a reason and at no risk to face disadvantages.

I consent to the collection, "pseudonymization", and password-protected storage of my study-related data on secure storage devices. Only authorized personnel of the Rudolf Frey Learning Clinic involved in this study will have access to this data. The raw data will not be shared with third parties or published.

Should the data be used for publication, it will be presented solely in an anonymized form, meaning that the data/results cannot be traced back to any individual. In accordance with scientific publication regulations, the data will be deleted after the retention period of 10 years.

I do not wish to participate in the study. ☐

I consent to participate in the study. ☐

A2. **Please enter your participation code:**

Section B: Baseline

B1. **Please indicate your position:**

Student ☐

Resident physician ☐

Specialist physician ☐

Senior physician ☐

Chief physician ☐

LimeSurvey

B2. **Please indicate your gender:**

- [ ] Male
- [ ] Female
- [ ] Non-binary

B3. **Please indicate your age:**

B4. **Please indicate your height [cm]:**

B5. **Please indicate your weight [Kg]:**

B6. **Please indicate your handedness:**

- [ ] Left handed
- [ ] Right handed
- [ ] Ambidextrous

B7. **What type of degree are you currently pursuing or have you completed?**

- [ ] Human medicin
- [ ] Dentistry
- [ ] Both

B8. **In which semester of your studies are you currently?**

(to be completed by students only)

B9. **Which specialization are you pursuing?**

(to be completed by medical professionals only)

B10. In which year of your advanced training are you currently?

(to be completed by medical professionals only)

B11. What is your current DEGUM level?

- None ☐
- DEGUM I ☐
- DEGUM II ☐
- DEGUM III ☐

Section C: Previous experience in sonography

C1. Have you completed an ultrasound course or continuing education in sonography?

- Yes ☐
- No ☐

C2. In which clinical areas have you conducted sonographic examinations?

(Multiple selections allowed)

- Head and neck sonography ☐
- Abdominal sonography ☐
- Cardiac sonography ☐
- Pulmonal sonography ☐
- Musculoskeletal sonography ☐
- Gynecological and obstetric ultrasound ☐
- Emergency sonography ☐
- Vascular sonography ☐

C3. How many sonographic examinations have you performed independently *(regardless of specialty)*?

An approximate estimation of the number of examinations is sufficient!

LimeSurvey

C4. **How many sonographic examinations have you performed independently in the following specialties:**

An approximate estimation of the number of examinations (adjusted to 100 examinations) is sufficient.

Head and neck sonography

Abdominal sonography

Cardiac sonography

Pulmonal sonography

Musculoskeletal sonography

Gynecological and obstetric ultrasound

Emergency sonography

Gefäßultraschall

C5. **How many years of experience do you have in the field of ultrasound diagnostics?**

(For less than one year, please specify the number of months as "X mon")

Section D: Current role in the field of ultrasound

D1. **How frequently have you used ultrasound as part of your clinical work currently/over the past 12 months?**

- Daily
- Several times a week
- Several times a month
- Rarely (Occasionally throughout the year)
- Never

D2. **How often do you currently perform an ultrasound examination per week?**

D3. **Which hand do you use to operate the ultrasound probe?**

- Primarily the left hand ☐
- Primarily the right hand ☐
- Both hands approximately equally often ☐

D4. **In which position do you primarily perform ultrasound examinations?**

- Primarily sitting ☐
- Primarily standing ☐
- Both positions approximately equally ☐

D5. **Which types of ultrasound examinations have you performed most frequently in the past 12 months?**

(Please select up to 2 options)

- Head and neck sonography ☐
- Abdominal sonography ☐
- Cardiac sonography ☐
- Pulmonal sonography ☐
- Musculoskeletal sonography ☐
- Gynecological and obstetric sonography ☐
- Emergency sonography ☐

Section E: Previous experience with ergonomics

E1. **Have you received instruction on general aspects of ergonomics as part of your medical training and/or education?**

- Yes ☐
- No ☐

E2. **Have you attended any specialized training on ergonomics in the workplace?**

- Yes ☐
- No ☐

E3. **If so, why did you attend the training?**

- Voluntarily ☐
- Mandatory training ☐
- To prevent work-related musculoskeletal disorders (WRMSD) ☐
- During WRMSD-Treatment ☐
- Other ☐

Other

[ ]

E4. **If so, in which year did the training take place?**

The indication of the year is entirely sufficient.

[| | | | | | | | |]

E5. **If so, what was the duration of the training *(in hours)*?**

[| | | | | | | | |]

E6. **If so, were the ergonomic aspects of performing ultrasound examinations also addressed?**

- Yes ☐
- No ☐

E7. **If you have completed an ultrasound course or continuing education, were specific aspects of ergonomics at the sonography workstation covered or taught?**

(Only to be answered if you have previously attended an ultrasound course or training)

- Yes ☐
- No ☐

Section F: Current status of ergonomics in the workplace

F1. **How important is appropriate ergonomic practice in your daily work?**

	1	2	3	4	5	6	7
Not important | Very important	☐	☐	☐	☐	☐	☐	☐

LimeSurvey

F2. **How would you describe your experience with the support provided by your employer regarding ergonomic measures?**

(Only to be answered if you are currently employed)

	1	2	3	4	5	6	7
Very bad | Very good	☐	☐	☐	☐	☐	☐	☐

F3. **Has your sonography workstation been specifically designed for ergonomic comfort?**

Yes ☐

No ☐

I don't know ☐

F4. **Which of the following supportive examination tools are available to you in your sonography workspace?**

(Multiple selections allowed)

Height-adjustable examination table ☐

Height-adjustable chairs ☐

Height-adjustable ultrasound machines ☐

Adjustable ultrasound monitors ☐

None of the above ☐

Other ☐

Other

[ ]

F5. **How often do you of these tools?**

	1	2	3	4	5	6	7
Never | Often	☐	☐	☐	☐	☐	☐	☐

F6. **How often do you take breaks during long ultrasound examinations to prevent physical discomfort?**

	1	2	3	4	5	6	7
Never | Often	☐	☐	☐	☐	☐	☐	☐

Section G: Motivation and Need for Ergonomics

G1. **How important do you think it is to adhere to ergonomic working practices at the workplace?**

	1	2	3	4	5	6	7
Not important I Very important	☐	☐	☐	☐	☐	☐	☐

G2. **Would you participate in a training course on general workplace ergonomics?**

	1	2	3	4	5	6	7
Not at all I Fully	☐	☐	☐	☐	☐	☐	☐

G3. **Would you attend a training/workshop on ergonomics in the specific context of performing ultrasound examinations?**

	1	2	3	4	5	6	7
Not at all I Fully	☐	☐	☐	☐	☐	☐	☐

G4. **Which ergonomic challenges have you experienced in your workplace in the past? Please describe them briefly:**

G5. **Do you have any suggestions for improving ergonomic practices in the workplace?**

Section H: Muscular-skeletal-disorders (specific)

Guidelines for Completion

This questionnaire addresses musculoskeletal trouble in ten different body regions. We kindly ask you to indicate whether you have ever experienced pain, ache or discomfort in each of these regions. The precise locations of these body regions are illustrated in the images provided on the following pages.

What is meant by trouble?

In this questionnaire, trouble refers to sensations such as stabbing pain, aches, discomfort or abnormal sensations (e.g., tingling, numbness) in the specified body regions. The trouble may be localized or diffuse. Please also consider trouble that radiates to other body regions (e.g., sciatica) or results in restricted movement.

H1. Does your physical trouble in the respective body parts seem to be connected to your ultrasound scanning activities?

(multiple responses allowed)

- Neck and cervical spine ☐
- Shoulder joints and upper arm ☐
- Elbow and forearm ☐
- Hand and wrists ☐
- Thoracic spine ☐
- Lumbar spine and lower back ☐
- Hip joints and thighs ☐
- Knee joints ☐
- Lower legs ☐
- Feet and ankle joints ☐

H2.

Have you at any time during the past 12 months had trouble (ache, pain, discomfort) in the neck region and/or cervical spine ?

- Yes ☐
- No ☐

H3. Have you experienced this trouble at any time in the past 7 days?

- Yes ☐
- No ☐

H4.

Have you at any time during the past 12 months had trouble (ache, pain, discomfort) in the shoulder joints and/or upper arms?

Yes ☐

No ☐

H5. **Have you experienced this trouble at any time in the past 7 days?**

Yes ☐

No ☐

H6. **Where did the trouble in the shoulder joints and/or upper arms predominantly occur?**

Right side ☐

Left side ☐

Both sides ☐

H7.

Have you at any time during the past 12 months had trouble (ache, pain, discomfort) in the elbows and/or forearms?

Yes ☐

No ☐

H8. **Have you experienced this trouble at any time in the past 7 days?**

Yes ☐

No ☐

H9. **Where did the trouble in the elbows and/or forearms predominantly occur?**

Right side ☐

Left side ☐

Both sides ☐

H10.

Have you at any time during the past 12 months had trouble (ache, pain, discomfort) in the hands and/or wrists?

Yes ☐

No ☐

H11. **Have you experienced this trouble at any time in the past 7 days?**

Yes ☐
No ☐

H12. **Where did the trouble in the hands and/or wrists predominantly occur?**

Right side ☐
Left side ☐
Both sides ☐

H13.

Have you at any time during the past 12 months had trouble (ache, pain, discomfort) in the thoracic spine?

Yes ☐
No ☐

H14. **Have you experienced this trouble at any time in the past 7 days?**

Yes ☐
No ☐

H15.

Have you at any time during the past 12 months had trouble (ache, pain, discomfort) in lumbar spine and/or lower back?

Yes ☐
No ☐

H16. **Have you experienced this trouble at any time in the past 7 days?**

Yes ☐
No ☐

H17.

Have you at any time during the past 12 months had trouble (ache, pain, discomfort) in hip joints and/or thighs?

Yes ☐
No ☐

H18. **Have you experienced this trouble at any time in the past 7 days?**

Yes ☐
No ☐

H19.

Have you at any time during the past 12 months had trouble (ache, pain, discomfort) in your knee joints?

Yes ☐

No ☐

H20. Have you experienced this trouble at any time in the past 7 days?

Yes ☐

No ☐

H21.

Have you at any time during the past 12 months had trouble (ache, pain, discomfort) in your lower legs?

Yes ☐

No ☐

H22. Have you experienced this trouble at any time in the past 7 days?

Yes ☐

No ☐

H23.

Have you at any time during the past 12 months had trouble (ache, pain, discomfort) in your feet and/or ankle joints?

Yes ☐

No ☐

H24. Have you experienced this trouble at any time in the past 7 days?

Yes ☐

No ☐
